# Caveolin-1 Is Essential for the Improvement of Insulin Sensitivity through AKT Activation during Glargine Treatment on Diabetic Mice

**DOI:** 10.1155/2021/9943344

**Published:** 2021-12-07

**Authors:** Hangya Peng, Panwei Mu, Haicheng Li, Shuo Lin, Chuwen Lin, Keyi Lin, Kunying Liu, Wen Zeng, Longyi Zeng

**Affiliations:** ^1^Department of Endocrinology and Metabolism, Guangdong Provincial Key Laboratory of Diabetology, The Third Affiliated Hospital of Sun Yat-Sen University, Guangzhou 510630, China; ^2^Ward 2 of Coronary Heart Diseases Centre, Fuwai Yunnan Cardiovascular Hospital, Kunming 650000, China

## Abstract

Insulin treatment was confirmed to reduce insulin resistance, but the underlying mechanism remains unknown. Caveolin-1 (Cav-1) is a functional protein of the membrane lipid rafts, known as caveolae, and is widely expressed in mammalian adipose tissue. There is increasing evidence that show the involvement of Cav-1 in the AKT activation, which is responsible for insulin sensitivity. Our aim was to investigate the effect of Cav-1 depletion on insulin sensitivity and AKT activation in glargine-treated type 2 diabetic mice. Mice were exposed to a high-fat diet and subject to intraperitoneal injection of streptozotocin to induce diabetes. Next, glargine was administered to treat T2DM mice for 3 weeks (insulin group). The expression of Cav-1 was then silenced by injecting lentiviral-vectored short hairpin RNA (shRNA) through the tail vein of glargine-treated T2DM mice (CAV1-shRNA group), while scramble virus injection was used as a negative control (Ctrl-shRNA group). The results showed that glargine was able to upregulate the expression of PI3K and activate serine phosphorylation of AKT through the upregulation of Cav-1 expression in paraepididymal adipose tissue of the insulin group. However, glargine treatment could not activate AKT pathway in Cav-1 silenced diabetic mice. These results suggest that Cav-1 is essential for the activation of AKT and improving insulin sensitivity in type 2 diabetic mice during glargine treatment.

## 1. Introduction

Insulin resistance plays an important role in the occurrence and development of type 2 diabetes mellitus (T2DM) [1]. Early intensive insulin therapy has been reported to delay or even partially restore the development of type 2 diabetes mellitus by improving insulin resistance (IR) [2, 3]. However, the underlying mechanism is still unclear.

Visceral adipose tissue is a vital insulin responsive organ and has been confirmed to be positively correlated with insulin resistance [4]. Thus, improving insulin responsiveness of visceral fat has been regarded as an effective way to alleviate insulin resistance. Caveolin (Cav-1) is a structural component of caveolae and was discovered in 1950s [5, 6]. Cav-1 is expressed abundantly in adipose tissue and is involved in many signaling pathways (e.g. insulin signaling) [7]. Caveolin-1 knockout mice develop remarkable insulin resistance [8, 9], while upregulation of caveolin-1 could enhance insulin signal transduction and therefore improve glucose uptake after insulin stimulation [10]. Besides, Cav-1 minor allele could predict an association between IR and body fat composition [11]. In our preliminary study, we found a reduction of caveolin-1 mRNA in the omental adipose tissue of overweight patients [12]. Taken together, these results suggest that Cav-1 is responsile for insulin sensitivity. Additionally, previous studies showed that Cav-1 was involved in the adipocyte differentiation process through the PI3K/AKT pathway and the development of IR [13, 14]. Thus, we hypothesized that insulin treatment would upregulate Cav-1 expression in adipose tissue to alleviate insulin resistance. In this study, we used lentivirus to silence Cav-1 in type 2 diabetic mice and investigated the effect of Cav-1 depletion on insulin sensitivity, PI3K p110 expression, and AKT activation after glargine treatment.

## 2. Material and Methods

### 2.1. Cav-1 Knockout Type 2 Diabetic Mouse Model

**Figure 1:**
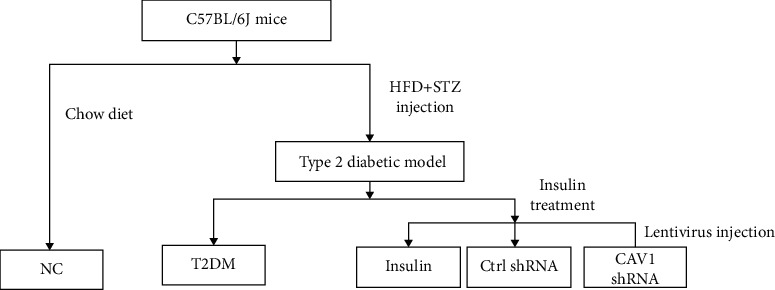
Flow chart of the experimental design.

Eight-week-old male C57BL/6J mice, 20-24 g, were purchased from the SLAC Animal Laboratory Company (Shanghai, China) and fed in the animal center at The Third Affiliated Hospital of Sun Yat-Sen University (Guangzhou, China). The animal containment room was conditioned at 20 °C - 25 °C and 50% ± 5% relative humidity with a 12 h light-dark cycle. A standard chow diet (4% fat (wt/wt), Guangdong Medical Laboratory Animal Center) was used to feed the negative control (NC) group (*n* = 5) and a high-fat diet (HFD) (60% fat, D12492, Research Diets) to feed the experimental group (*n* = 20) (the feeding condition continues till the end of experiment). After 4 weeks of HFD feeding, the proposed diabetic group (*n* = 20) was injected intraperitoneally with a low dose streptozotocin (STZ, 40 mg/kg) (Sigma, USA) for 3 consecutive days after overnight fasting. Two weeks after the last injection of STZ, intraperitoneal glucose tolerance test (IPGTT) and intraperitoneal insulin tolerance test (IPITT) were performed to determine whether the diabetic mice were successfully set up. The diabetic mice were then randomly randivided into 4 groups (*n* = 5 for each group): T2DM (without any treatment), insulin (receiving insulin treatment), CAV-1-shRNA (receiving insulin treatment after Cav-1 silencing), and Ctrl-RNA (receiving insulin treatment without Cav-1 silencingd) (Figure 1). In the CAV-1-shRNA group, Cav-1 was silenced by injection of lentivirus via the tail vein. In the Ctrl-RNA group, the mice were injected vectors through the tail vein. All the insulin-treated groups were injected subcutaneously with glargine, starting with 0.4 u/d and then adjusted to the amount of maintaining their fasting blood glucose at the same level for 2 weeks (14 days). The body weight of mice was measured biweekly, while their fasting blood glucose was monitored weekly during the whole process. At the end of the experiment (on the 15^th^ day of insulin treatment), all mice were fasted for 8 hours, anaesthetised by ether, and sacrificed for blood and periepididymal adipose tissue collection. All procedures were carried out in accordance with National Institutes of Health guidelines and approved by the Institute Animal Care and Use Committee (IACUC) at the Animal Ethics Committees of the Sun Yat-Sen University.

### 2.2. CAV-1 Knockout Diabetic Mice

CAV-1 was silenced by targeted-Cav-1 lentivirus (Lv) injection with short hairpin RNA (shRNA), which was designed and synthesized by Shanghai GeneChem Co., Ltd. (Shanghai, China). The sequence of targeted-Cav-1-RNAi used was ACGTGGTCAAGATTGACTT. Lentiviral vectors with green fluorescence protein (GFP) tag were used as the vector controls of RNA inteference (Ctrl-RNA). Each mouse was injected with 5∗10^7^ transfection units of lentiviruses.

### 2.3. Intraperitoneal Glucose Tolerance Test and Intraperitoneal Insulin Tolerance Test

In the intraperitoneal glucose tolerance test (IPGTT), mice were administered with glucose (2 g/kg wt i.p.) after overnight fasting. The intraperitoneal insulin tolerance test (IPITT) was conducted by insulin injection (0.75 units/kg wt i.p.) (Novolin R; Novo Nordisk, Denmark) after 6 hours of fasting (7 am-1 pm). Tail vein blood glucose level was measured at 0, 30, 60, and 120 min by the OptiumXceed glucometer (Abbott Diabetes Care, Inc., Alameda, CA). The methods were confirmed elsewhere [15]. To avoid hypoglycemia, the fasting periods for IPGTT and IPITT were different. For IPGTT, mice were usually fasted overnight. But for IPITT, the fasting period was shorter, usually 6 hours [15, 16] or even 4 hours [17]. Here, we adopted overnight fasting for IPGTT and 6 hours fasting for IPITT, respectively.

### 2.4. Western Blotting

Periepididymal adipose tissues were collected and stored at -80 °C for western blot analysis. For protein isolation, periepididymal adipose tissues were homogenized with whole cell lysis buffer, protease inhibitor, and phosphatase inhibitor. The primary antibodies were purchased from Cell Signaling Technology (Danvers, MA, USA), which included Caveolin-1-Ab (Cat. #3267), phosphorylated (p)-Akt (Ser473)-Ab (Cat. #4060, 1 : 2000), Akt-Ab (Cat. #4691), PI3K-Ab (Cat. #4255), and *β*-actin (Cat. #8457). All primary antibodies were diluted 1000 folds, unless otherwise indicated. After incubation with primary antibodies for 24 h at 4°C, membranes were further incubated with secondary antibodies DyLight 800 (1 : 10000) (Thermo Fisher Scientific) for 1 h at room temperature. Finally, the membranes were then scanned and captured with the Odyssey Infrared Imaging System (LI-COR Biosciences, Lincoln, NE). Bands were quantified using the ImageJ software.

### 2.5. Immunofluorescence Analysis

For immunofluorescent analysis, the frozen paraepididymal tissue cut from cross sections (10 *μ*m) was blocked with 10% BSA in PBS for 60 min at room temperature and washed three times with PBS for 5 min. Primary antibodies targeting Caveolin-1 (Cat. #ab211503, goat anti-caveolin-1, Abcam, dilution 1 : 50) and GLUT4 (Cat. #ab33780, rabbit anti-glut4, Abcam, dilution 1 : 50) were applied overnight at 4°C. Next, coverslips were washed with PBS and incubated with appropriate fluorescent-labeled secondary antibodies (Donkey anti-Goat IgG (H + L) Highly Cross-Adsorbed Secondary Antibody, Alexa Fluor Plus 488, Cat. #A32814, Goat anti-Rabbit IgG (H + L) Highly Cross-Adsorbed Secondary Antibody, Alexa Fluor Plus 594, Cat. #A32740, Invitrogen, Thermo Fisher Scientific, dilution 1 : 500) for 1 h at room temperature. Cell nuclei were then stained with DAPI (1 *μ*g/mL, Roche) for 5 min and washed with PBS, and the resulting slides were mounted with Prolong Gold Antifade Mountant (Life Technologies). Images of samples were obtained using fluorescence microscopy (DMI8, Leica, Germany). The intensity of fluorescence was calculated by the ImageJ software.

### 2.6. Statistical Analysis

Data were presented as mean ± SEM. One way ANOVA test was performed to determine whether there were significant differences between groups. Multiple comparisons between groups were analyzed with LSD analysis of variance. *P* < 0.05 was considered to be statistically significant.

## 3. Results

### 3.1. Type 2 Diabetic Mice Developed Insulin Resistance and Glucose Intolerance

**Figure 2:**
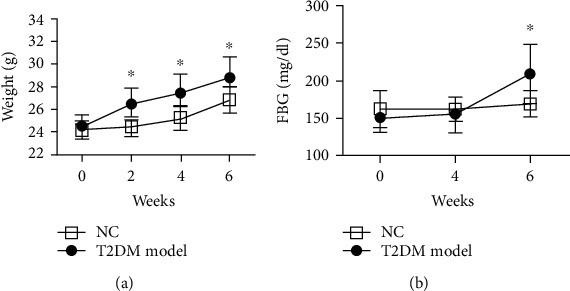
Comparison of weight and FBG between NC and T2DM groups. Body weight of T2DM mice increased significantly after HFD feeding for two weeks (a). FBG was elevated in the T2DM group after STZ injection for two weeks (b) (data presented as mean ± SEM. ^∗^*P* < 0.05 vs. NC mice, *n* = 5).

**Figure 3:**
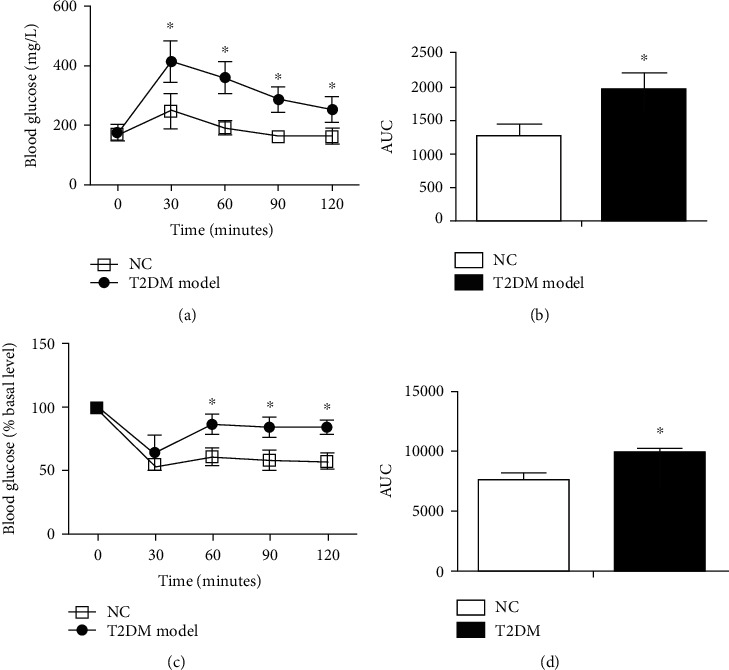
Blood glucose levels and AUCs of blood glucose in NC and T2DM group after IPGTT (a, b) and IPITT (c, d) (data presented as mean ± SEM. ^∗^*P* < 0.05 vs. NC mice, *n* = 5).

Compared with the NC group, the body weight (24.44 ± 0.82 g vs. 26.44 ± 1.40 g) (Figure 2(a)) and blood glucose (BG) (168.94 ± 16.62 mg/dL vs. 209.18 ± 35.60 mg/dL) (Figure 2(b)) of mice in the T2DM model group increased significantly after two weeks of HFD feeding and administration of STZ, respectively. Impaired insulin sensitivity was verified by IPITT. As displayed by the area under the curve (AUC) of graph in Figure 3(b), the blood glucose between NC and T2DM model group were 1275.00 ± 178.13 vs. 1969.89 ± 255.28 (*P* < 0.05). The impaired glucose tolerance of mice was verified by IPGTT. The AUC of BG in Figure 3(d) between NC and T2DM model group were 7577.13 ± 614.93 vs. 9839.52 ± 426.32 (*P* < 0.05).

### 3.2. Glargine Upregulates the Expression of PI3K p110, AKT Phosphorylation and Caveolin-1 in Periepididymal Adipose Tissue

**Figure 4:**
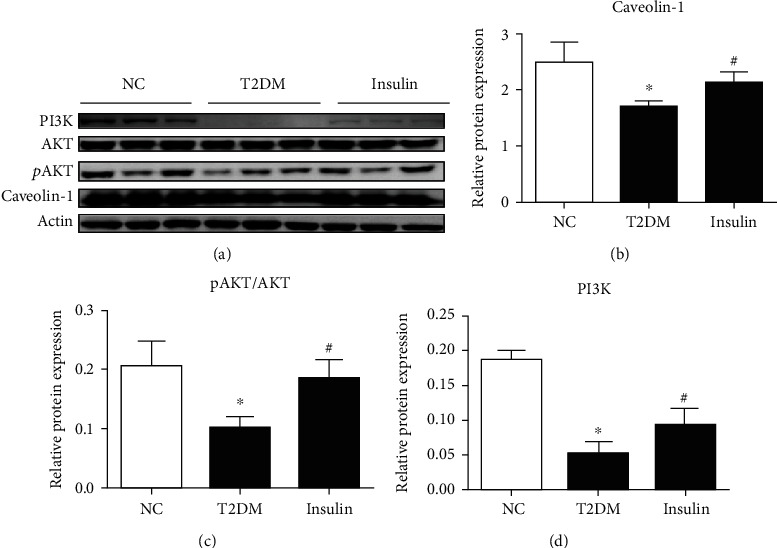
The expression of Caveolin-1, p-AKT/AKT, and PI3K in adipose tissue of NC, type 2 diabetic mice, and insulin-treated T2DM mice (a). The band intensity of Caveolin-1, p-AKT/AKT, and PI3K was quantified as shown in (b–d), respectively (data presented as mean ± SEM. ^∗^*P* < 0.05 vs. NC mice. #*P* < 0.05 compared with T2DM mice, *n* = 5).

After development of type 2 diabetes, mice in the insulin group were treated with glargine for 2 weeks. Compared with the mice in the NC group, mice in the T2DM group had lower levels of PI3K p110, a reduction in AKT phosphorylation, and caveolin-1 was downregulated in their periepididymal adipose tissue. However, after 2 weeks of glargine administration, PI3K p110 and p-AKT levels in T2DM groups were increased, and caveolin-1 expression was upregulated (Figure 4).

### 3.3. The Expression of PI3K p110 and AKT Phosphorylation Could Not Be Upregulated after Caveolin-1 Depletion Even with Treatment of Glargine

**Figure 5:**
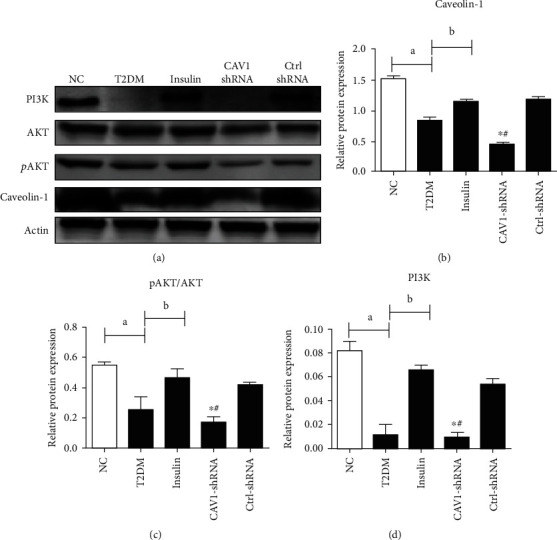
The expression of Caveolin-1, p-AKT/AKT, and PI3K among five different groups (a). The band intensity of Caveolin-1, p-AKT/AKT, and PI3K was quantified as shown in (b–d), respectively (data are expressed as mean ± SEM. ^∗^*P* < 0.05 compared with the insulin group. #*P* < 0.05 compared with the Ctrl-shRNA group; a represents *P* < 0.05 compared with the NC group, and b represents *P* < 0.05 compared with the T2DM group, *n* = 5).

In order to address the role of caveolin-1 in glargine-stimulating PI3K p110 expression and AKT activation, CAV-1 was silenced by short hairpin RNA in diabetic mice (CAV-1-shRNA group). Lentivirus vector without CAV-1 sequence was used as RNA inference control (Ctrl-RNA group). Glargine was administrated to the CAV-1-shRNA group, Ctrl-RNA group, and insulin group for 2 weeks. The inhibition of caveolin-1 expression led to a reduction in PI3K p110 and p-AKT expression in the CAV-1 shRNA group mice compared with the Ctrl-RNA and insulin group. (Figure 5).

### 3.4. Higher Glargine Doses Were Required to Maintain Fasting Blood Glucose in CAV-1 Silenced Diabetic Mice

**Figure 6:**
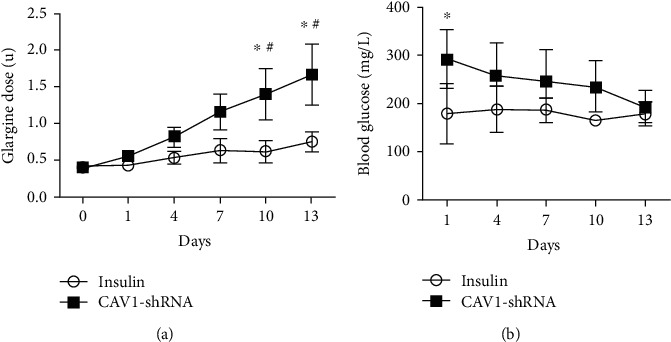
The doses of glargine required to maintain blood glucose in the CAV1-shRNA and insulin groups (a). The fluctuation of blood glucose in the CAV1-shRNA and insulin groups (b) (data are expressed as mean ± SEM. ^∗^*P* < 0.05 compared with the insulin group, *n* = 5).

During the course of glargine treatment, the starting dose administered was 0.4 u/d and then adjusted ±0.1 u/d to maintain the fasting blood glucose between 72 mg/dL and 144 mg/dL [18]. Higher doses of glargine were administered, ranging from 0.4 u/d to 1.6 u/d in the CAV-1-shRNA group, while the insulin group received stable doses between 0.4 u/d and 0.8 u/d (Figure 6(a)). On day 1 after glargine treatment, the fasting blood glucose (FBG) between these two groups was markedly different (178.97 ± 61.80 mg/dL vs. 291.60 ± 60.62 mg/dL, *P* < 0.05). On the 4^th^ day of treatment, FBG was no longer significantly different between these two groups (187.5 ± 48.0 mg/dL vs. 257.4 ± 69 mg/dL) (Figure 6(b)). Glargine therapy narrowed the gap of FBG between these two groups. However, compared with the insulin group, the CAV-1-shRNA group required more glargine to lower blood glucose. In line with the glargine requirement to maintain the fasting blood glucose, the CAV-1-shRNA group had a higher HOMA-IR than the insulin group (24.802 ± 4.688 vs. 16.615 ± 2.407, *P* < 0.05). These results suggest that the CAV-1-shRNA group experienced worse insulin resistance than the insulin group.

### 3.5. The Translocation of GLUT4 Was Reduced in CAV1-shRNA Group

**Figure 7:**
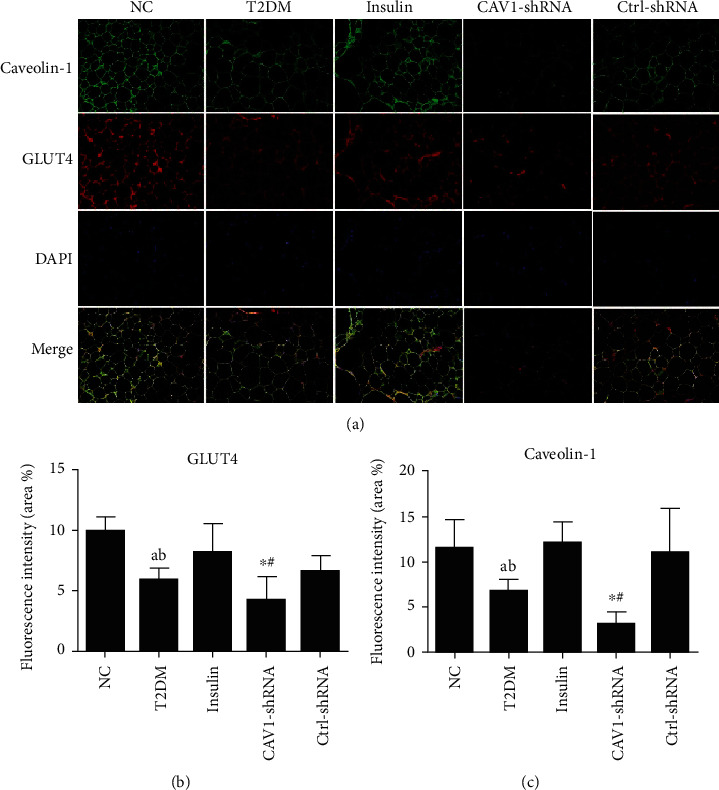
Immunofluorescence results of the visceral adipose tissue from all five groups. Representatives of fluorescent microscope images stained with Caveolin-1 (green), GLUT4 (red), and DAPI (blue). Images of the adipose tissue were taken using a microscope under 20x objective lens (data are expressed as mean ± SEM. ^∗^*P* < 0.05 compared with the insulin group. #*P* < 0.05 compared with the Ctrl-shRNA group, a represents *P* < 0.05 compared with the NC group, and b represents *P* < 0.05 compared with the insulin, *n* = 5).

The translocation of GLUT4 in the plasma membrane of periepididymal adipose tissue was investigated by immunofluorescence (Figure 7). Compared with the NC group, type 2 diabetic mice were presented with reduced Caveolin-1 (green) and GLUT4 (red) fluorescence intensity (fluorescence intensity of Caveolin-1: 11.46 ± 3.29 vs. 6.74 ± 1.35, GLUT4: 9.915 ± 1.29 vs. 5.87 ± 0.98). But after the treatment of insulin (insulin group), the fluorescence intensity of Caveolin-1 and GLUT4 was enhanced compared with the T2DM group (fluorescence intensity of Caveolin-1: 12.08 ± 2.35 vs. 6.74 ± 1.35, GLUT4: 8.12 ± 2.44 vs. 5.87 ± 0.98). However, as the expression of Caveolin-1 was silenced (CAV1-shRNA group), the fluorescence intensity of GLUT4 was reduced even with insulin treatment compared with the Ctrl-shRNA group (fluorescence intensity of Caveolin-1: 3.08 ± 1.40 vs. 10.99 ± 3.98, GLUT4: 4.19 ± 1.98 vs. 6.56 ± 1.35).

## 4. Discussion

T2DM has become a global concern over the past three decades, accounting for more than 90% of individuals diagnosed with diabetes [19, 20]. Attenuation of insulin metabolic action plays a vital role in the development of T2DM [21]. Thus, improving insulin sensitivity has become a recognized way to treat T2DM patients. Hu et al. showed that insulin sensitivity of patients with T2DM had been improved after insulin treatment as demonstrated by lower HOMA-IR values compared with normal glucose tolerance people [2]. But the mechanism is still unknown. In the molecular biology researches of insulin resistance and metabolism, caveolin-1 has been studied since early 1990s [22]. In the current study, we found that caveolin-1 could play a vital role in alleviating insulin resistance during insulin treatment by activating AKT phosphorylation.

Caveolae are 50-100 nm invaginations found within the plasma membrane of cells. It involves in many bioprocesses that are essential for homeostasis, most notably endocytosis, mechano-protection, and signal transduction [23]. Caveolin-1, the major scaffolding protein of caveolae (21-24 kDa), can recruit and regulate various signaling proteins through the interation of its scaffolding domain with hydrophobic sequence [24]. It has been demonstrated that Cav-1 can assist Akt signaling in mechanotransduction of vascular smooth muscle cells [25], indicating the close relationship between Cav-1 and AKT signaling. As a classical pathway in insulin signal transduction, any defects in the PI3K p110 and AKT activation along with the downstream molecules will lead to insulin resistance [26–28]. Consistent with these studies, our study showed that the expression of PI3K p110 and AKT phosphorylation were lowered in visceral adipose tissue of T2DM mice. Additionally, we found a lower expression of caveolin-1 in periepididymal adipose tissue of diabetic mice when compared to the NC and insulin groups. Similar results (the reduced expression of caveolin-1) have been reported in the omental adipose tissue of individuals who were overweight or had obesity [12, 29]. However, the lowered expression of caveolin-1 in diabetic mice was not observed in subcutaneous fat (Supplementary Fig. 1). This discrepancy may be due to the different function of intra-abdominal fat and subcutaneous fat. Intra-abdominal fat is strongly and independently associated with both insulin resistance and poor glycemic control [30], while the subcutaneous adipose tissue is thought to maintain insulin sensitivity [31]. Thus, this suggests that downregulation of caveolin-1 in periepididymal adipose tissue can contribute to the pathogenesis of obesity and insulin resistance.

Schultze et al. revealed that glargine could activate the PI3K/AKT pathway and lower the blood glucose in humans and mice [32]. In accordance with these findings, our results showed an increase in p110 PI3K expression and AKT phosphorylation in periepididymal white adipose tissue of mice after receiving glargine. In addition, it was observed that the expression of caveolin-1 in T2DM was also increased after the glargine treatment. These results suggests that upregulation of caveolin-1 may be involved in activating AKT phosphorylation during insulin treatment. In order to address this speculation, CAV-1 was silenced by lentivirus with hairpin RNA in diabetic mice and then treated with glargine (CAV-1-shRNA group). The results showed that Akt activation could not be improved by glargine treatment when caveolin-1 was silenced. Moreover, the CAV-1-shRNA group expressed lower levels of PI3K p110 expression and p-AKT compared with the Ctrl-shRNA group, which implied the impairment of AKT activation in caveolin-1 absent mice. Taken together, these results suggest that caveolin-1 is indispensable to the insulin-activated AKT phosphorylation.

During treatment, the CAV-1-shRNA group required more glargine to maintain blood glucose level compared to the insulin group (Figure 6). This suggested that insulin resistance was more severe in caveolin-1 silenced mice than mice in the control group. HOMA-IR was higher in CAV-1-shRNA mice than that in the insulin group (Table 1). Additionally, CAV1-shRNA mice presented the highest serum insulin levels (555.999 ± 40.033 pmol/L) of all the groups (141.753 ± 24.408 pmol/L in the NC group, 246.112 ± 46.055 pmol/L in the T2DM group, 193.957 ± 67.549 pmol/L in the insulin group, 293.829 ± 58.344 pmol/L in the Ctrl-RNA group), which indicated a worsened insulin resistance (Supplementary Fig. 2). This may be due to the reduction of caveolin-1 blunted insulin-triggered GLUT4 recruitment (Figure 7) and insulin receptor stabilization in adipocytes [33, 34].

High-fat diet would interfere in lipid metabolism and exacerbate insulin resistance. Previous studies have demonstrated that caveolin-1 is involved in this process [35]. Caveolin-1 null mice were lean and resistant to diet-induced obesity due to reduced/atrophic fat deposits [9]. Histologically, when fed with HFD, Caveolin-1 knockout mice showed reduced adiposity, decreased expression of leptin and adiponectin compared with wild-type mice [9]. In our study, after feeding HFD for 8 weeks in total, the diameter of adipocytes in the Ctrl-RNA group was larger than the CAV1-shRNA group (Supplementary Fig. 3). This is similar with the previous study that caveolin-1 downregulation would result in the shrinkage of lipid droplet. Even with the atrophy of adipose tissue, CAV-1 null mice showed insulin resistance in our study. The reason might be that exacerbation of hyperphagia driven by low leptin levels leads to the harmful spillover of lipids to other insulin sensitive tissues [9, 36], leading to inflammation of these tissues such as muscles and liver. Thus, the lower expression of caveolin-1 could severe insulin resistance by affecting lipid metabolism.

In the future, further investigations are required to enrich our study. Firstly, more accurate methods can be used. For instance, a glucose clamp may be used to assess insulin resistance in diabetic mice before and after insulin treatment. Secondly, apart from silencing caveolin-1, the effect of caveolin-1 overexpression should also be applied. Thirdly, the lentivirus shRNA Cav-1 was not specific for adipose tissue; it was injected by tail vein, so it would silence Cav-1 expression in all the tissues of the mice. Thus, the effect of other insulin sensitive organs such as muscles and liver should be considered. And specific virus could be used to avoid the influence of other tissues. Finally, both *in vitro* and *in vivo* studies would be favorable to elucidate more underlying mechanisms, e.g. PI3K inhibitors need to be applied to further investigate caveolin's rolein PI3K/AKT pathway.

In conclusion, our study showed that caveolin-1 depletion could impair insulin sensitivity, PI3K p110 expression, and AKT activation. These findings suggest that CAV-1 has the potential to be a therapeutic target for the treatment of type 2 diabetes, obesity, and other metabolic disorders involving insulin resistance.

## Figures and Tables

**Table 1:** HOMA-IR of all groups at the end of the experiment.

Groups	NC	T2DM	Insulin	CAV1-shRNA	Ctrl-shRNA
HOMA-IR	10.807±1.622	18.123±2.122	16.615±2.407∗	24.802±4.688#	19.138±4.973

Data are expressed as mean ± SEM.∗P < 0.05 compared with T2DM #P<0.05 compared with Insulin group.

## Data Availability

The data used to support the findings of this study are available from the corresponding author upon request.

## Supplementary Materials

Supplementary Materials Supplementary Figure 1. Caveolin-1 protein expression (A) and quantification (B) in subcutaneous fat of NC, T2DM and Insulin group mice. Supplementary Figure 2. The fasting plasma insulin levels in five groups after 2 weeks glargine treatment. (Data are expressed as mean ± SEM. ^∗^ P<0.05 compared with Insulin group .^#^ P<0.05 compared with Ctrl-shRNA group , ^a^ represents P<0.05 compared with NC group, ^b^ represents P<0.05 compared with T2DM group, n=5). Supplementary Figure 3. H&E staining of periepdidymal adipose tisse from Ctrl-shRNA and CAV1-shRNA group.
