# Supplementary material for: Caveolin-1 Is Essential for the Improvement of Insulin Sensitivity through AKT Activation during Glargine Treatment on Diabetic Mice
Source: J Diabetes Res. 2021 Dec 7;2021:9943344. doi: 10.1155/2021/9943344 (PMC8670926; doi:10.1155/2021/9943344)
Supplement: Supplementary Materials — Supplementary Figure 1. Caveolin-1 protein expression (A) and quantification (B) in subcutaneous fat of NC, T2DM and Insulin group mice. Supplementary Figure 2. The fasting plasma insulin levels in five groups after 2 weeks glargine treatment. (Data are expressed as mean ± SEM. ∗ P<0.05 compared with Insulin group .# P<0.05 compared with Ctrl-shRNA group , a represents P<0.05 compared with NC group, b represents P<0.05 compared with T2DM group, n=5). Supplementary Figure 3. H&E staining of periepdidymal adipose tisse from Ctrl-shRNA and CAV1-shRNA group. [file 9943344.f1.zip › supplementary figure 1.docx]

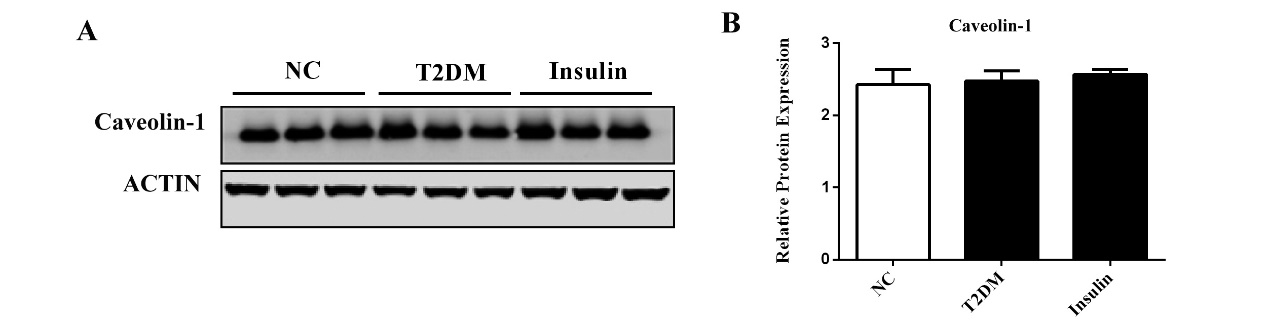


**Supplementary Figure 1.** Caveolin-1 protein expression(A) and quantification(B) in subcutaneous fat of NC, T2DM and Insulin group mice.
